# Plasma MiRNA alterations between NSCLC patients harboring Del19 and L858R EGFR mutations

**DOI:** 10.18632/oncotarget.10829

**Published:** 2016-07-24

**Authors:** Yihan Ma, Peiqi Xu, Yanjun Mi, Wenyi Wang, Xiaoyan Pan, Xiaoting Wu, Qi He, Hongming Liu, Weiwei Tang, Hanxiang An

**Affiliations:** ^1^ Xiamen Cancer Hospital, The First Affiliated Hospital of Xiamen University, 361003, Fujian, China; ^2^ Reproduction Center, The Second Affiliated Hospital of Kunming Medical University 650101, Yunnan, China

**Keywords:** circulating miRNA, microarray, NSCLC, EGFR, response

## Abstract

Based on recognition of driver mutations, treatment paradigm for non-small-cell lung cancer (NSCLC) patients has been shifted. However, recently exon 19 deletion mutation (del19) of epidermal growth factor receptor (EGFR) clearly shows better clinical benefit over single-point substitution mutation L858R in exon 21 (L858R). The aim of this study was to investigate the difference by analyzing the expression of plasma microRNAs (miRNAs) of NSCLC patients with EGFR mutation del19 or L858R. MiRNA microarray of plasma from patients' blood identified 79 mapped, network-eligible miRNAs (fold > 5), of which 76 were up regulated and 3 were down regulated. Genetic network was performed with Ingenuity Pathway Analysis (IPA). Among analysis, MYC, Argonaute2 (AGO2), Y-box binding protein 1 (YBX1), cyclin E1 (CCNE1) were involved in organismal abnormalities and cancer. Our findings provide information on the epigenetic signature of the two major sensitive mutations among NSCLC and add to the understanding of mechanisms underlying the different outcomes.

## INTRODUCTION

Being the leading cause of cancer mortality, lung cancer accounts for almost one third of all death of cancer, of which 75% are NSCLC [1]. Traditional cytotoxic chemotherapy, which is widely applied, offers a modest increase in survival but is also restricted by significant toxicity and low response rate among 20%–35% [2]. Activating mutations in EGFR confer hypersensitivity to tyrosine kinase inhibitors (TKI) in NSCLC patients, among which del19 and L858R are the two major classic mutations. Both mutations are consistently associated with dramatic and usually long-lasting response to TKI [3–5]. However, different publications reported that del19 seemed to have better clinical outcome than L858R [6, 7]. Recently, new statistics also indicated that del19 clearly exhibited more beneficial while L858R seemed equal to chemotherapy in overall survival (OS) [8–10]. However, the mechanisms about the difference remain unclear up to date.

MiRNAs are a type of small non-coding single-stranded RNAs with 20–25 nt length, typically regulating gene expression. Their critical roles in the development of various tumors have been widely reported. To some extent, miRNA microarray offers more information than traditional mRNA profiling [11, 12].

In the present study, we investigated the global expression profile of miRNAs from NSCLC patients' plasma using high-density miRNA microarray containing 3100 capture probes covering all human microRNAs annotated in miRBase 18.0. Interactive networks and gene functions were analyzed using the IPA tool to identify their corresponding mechanisms.

## RESULTS

### Patient characteristics

The characteristics of patients enrolled in this study were summarized in Table 1. There was no significant difference in the distribution of age, gender, clinical stage and smoking history between the patients with EGFR mutation del19 or L858R.

**Table 1:** Clinical characteristics of patients with EGFR mutation del19 and L858R

Characteristics	Del19	L858R	*P*
**Age (mean ± SD)**	54 ± 8.5	58 ± 7.7	> 0.05
**Sex**			> 0.05
Male	5	4	
Female	6	7	
**Smoking status**			> 0.05
Smoking	6	4	
None or little	5	7	
**EGFR TKIs**			> 0.05
Erlotinib	6	6	
Gefitinib	5	5	
**Treatment line**			> 0.05
First	10	9	
Second	1	2	

### Identification of differently expressed miRNAs between del19 and L858R mutations

To search differentially expressed miRNAs between EGFR del19 and L858R mutations, we performed the global expression profile of patients' plasma using high-density miRNA microarray containing all human microRNAs annotated. Microarray analysis revealed del19 mutation patients had a significant alteration expression in 79 miRNAs (fold > 5) compared with L858R mutation patients. 76 miRNAs were up-regulated and 3 miRNAs were down-regulated (Supplementary Table S1).

### Network and molecular function analyses

**Figure 1:**
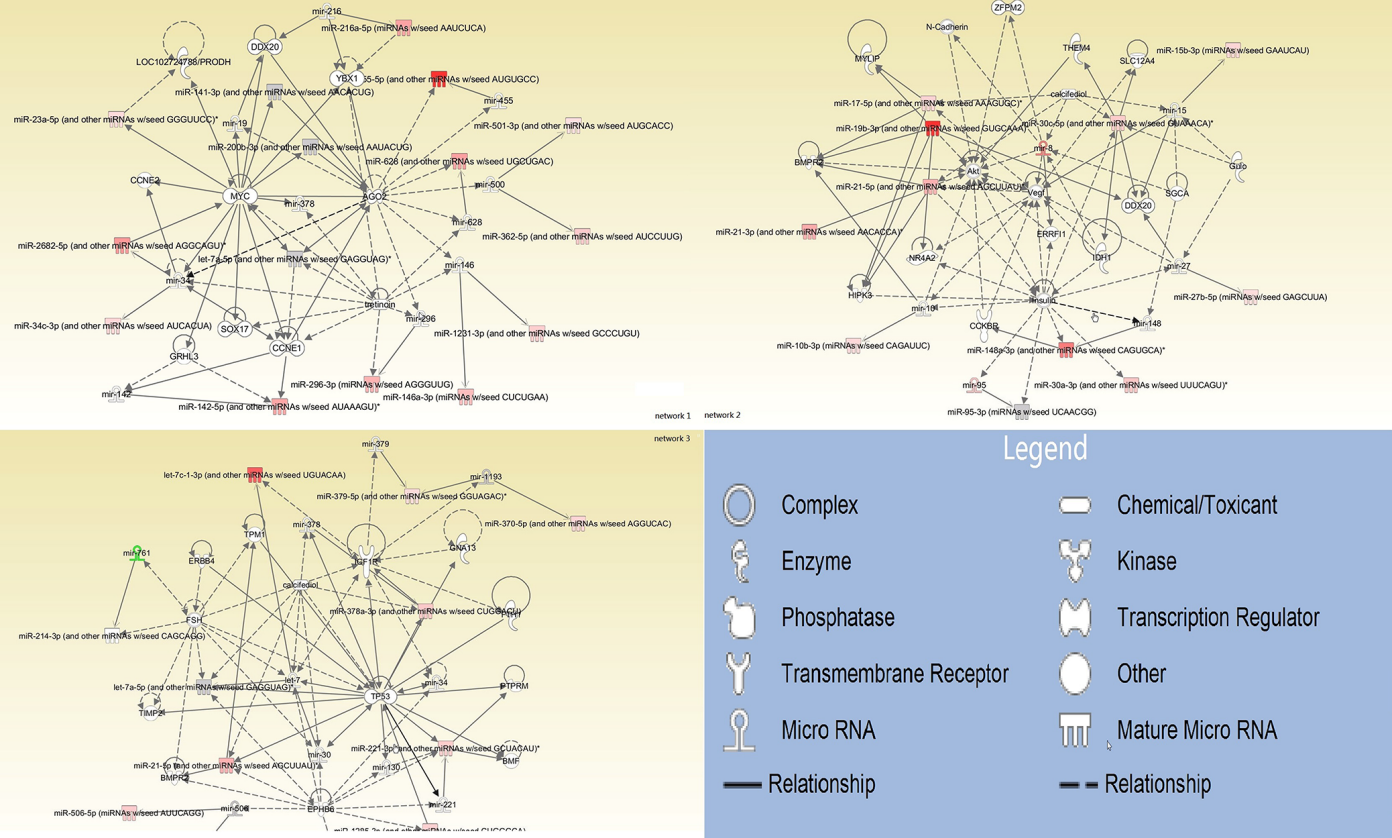
Interrelated networks of genes and miRNAs whose expression was different between del19 and L858R mutations In total, three important networks of interrelated miRNAs and target genes were identified. The three networks were merged by overlapping miRNAs.

79 miRNAs were mapped, network-eligible and classified into genetic networks. Among the 79 miRNAs, 62 shared at least one overlapping gene in common and 3 networks were merged together (Figure 1). IPA also depicted important biological pathways associated with these 3 merged networks (Table 2). Meanwhile, different molecular events directly related to cancer were identified (i.e. cell death and survival, cellular development, cellular growth and proliferation) (Table 3). Furthermore, their relationships with diseases and disorders were also assessed (Table 4).

**Table 2:** Interactive networks

Network	Molecules	Functions	Score
1	AGO2, CCNE1,CCNE2,DDX20,GRHL3,let-7a-5p,mir-141-3p,mir-142-5p,mir-146a-3p,mir-200b-3p,mir-216a-5p,mir-23a-5p,mir-2682-5p,mir-296-3p,mir-34c-5p,mir-362-5p,mir-455-5p,mir-5-1-3p,mir-628,MYC,SOX17,tretinoin,YBX1	Organismal injury and abnormalities Cancer	23
2	Akt,BMPR2,calcifediol,CCKBR,DDX20,ERRFI1,Gulo,HIPK3,IDH1,Insulin,mir-8,mir-10,mir-15,mir-27,mir-95,mir-148,mir-10b-3p,mir-148a-3p,mir-15b-3p,mir-17-5p,mir-19b-3p,mir-21-3p,mir-21-5p,mir-27b-5p,mir-30a-3p,mir-30c-5p,mir-95-3p,MYLIP,N-cadherin,NR4A2,SGCA,SLC12A4,THEM4,Vegf,ZFPM2	Cancer Reproductive system disease	22
3	BMF,BMPR2,calcifediol,EPHB6,ERBB4,FSH,FTH1,GNA13,IGF1R,Let-7,let-7a-5p,let-7c-1-3p,mir-30,mir-34,mir-130,mir-221,mir-378,mir-379,mir-506,mir-761,mir-1193,mir-1285,mir-1285-3p,mir-21-5p,mir-214-3p,mir-221-3p,mir-370-5p,mir-378a-3p,mir-379-5p,mir-506-5p,mir-514a-3p,PTPRM,TIMP2,TP53,TPM1	Developmental disorder Hereditary disorder	17

Network-eligible, overlapping genes (*n* = 79) whose expression was modified differentially between del19 and L858R mutational patients. Of these 79 differentially expressed miRNAs, 52 miRNAs was divided into three main networks and 10 miRNAs in other networks identified (data not shown). The rest of the genes either did not show any significant change or were not detected from the array.

**Table 3:** Molecular and cellular functions

Molecular and cellular functions	Molecules
Cell Death and Survival	7
Cellular Development	11
Cellular Movement	6
Cellular Growth and Proliferation	10

Molecular events directly related to cancer were identified through candidate miRNAs.

**Table 4:** Diseases and disorders

Diseases and disorders	Molecules
Organismal injury and abnormalities	25
Reproductive system disease	16
Connective tissue disorders	8
Inflammatory disease	9
Inflammatory response	9

Diseases and disorders directly related to candidate miRNAs founded through IPA.

The other 17 miRNAs which were highly expressed in del19 mutated patients were detected in separate networks without overlapping due to the lack of commonly-shared gene.

### Validation of microarray data by real-time PCR

**Figure 2:**
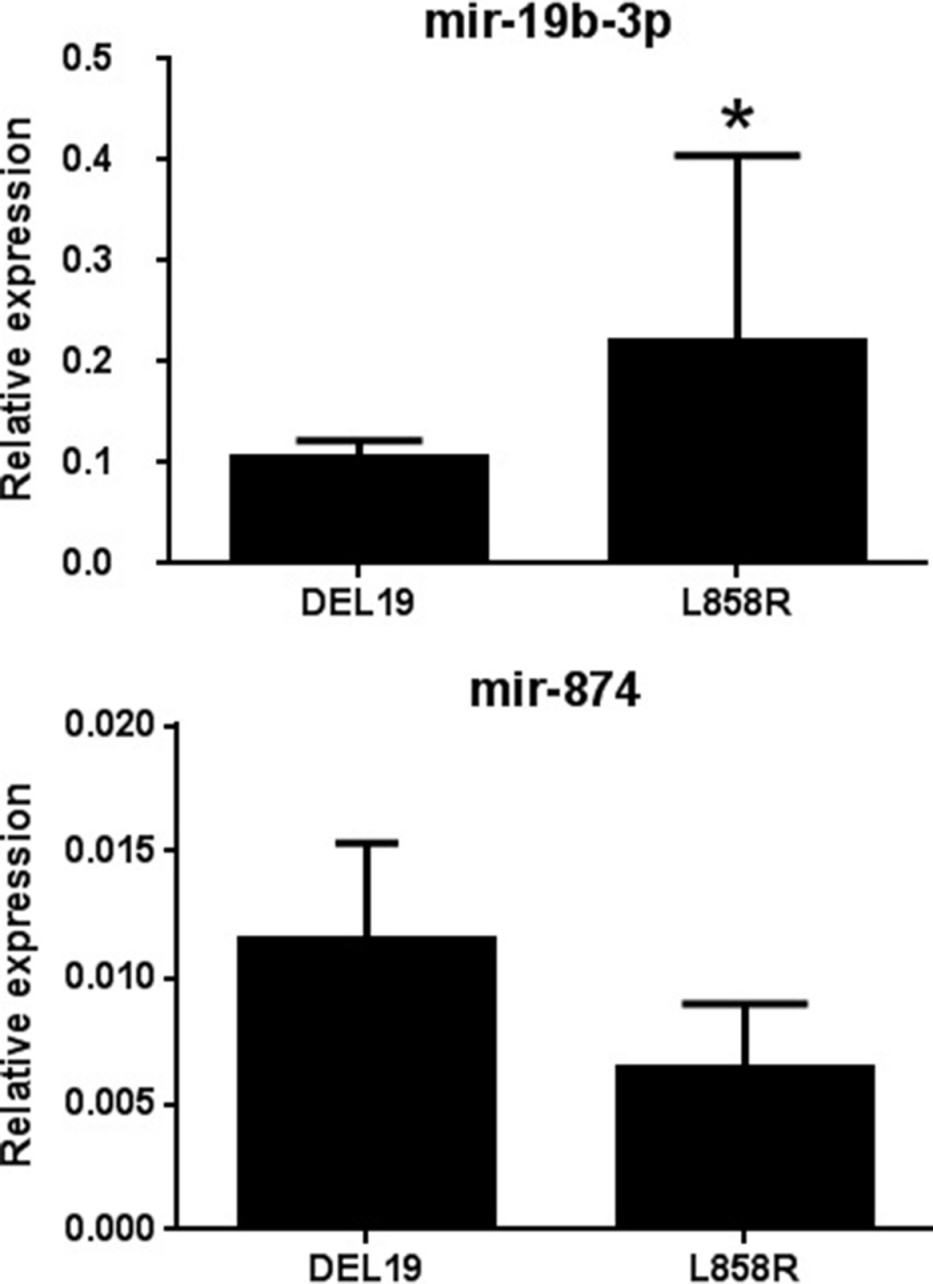
Validation of selected candidate differentially expressed miRNAs using qRT-PCR The results of transcriptional expression were normalized to the value of internal control (cel-mir-39). The relative expression was shown as mean ± SD. *Statistical significance of differences was analyzed by Student's test (*p* < 0.05).

Signaling pathway and network analyses revealed that miR-19b-3p and miR-874 regulate several important targets (DDX20, Akt, and NR4A2; AGO1, TAOK2, and E2F3, respectively) involved in tumorgenesis and progression. These two differentially expressed miRNAs were selected and verified by qRT-PCR using miRNA purified from the same patients used for the microarray experiment. The relative expression levels of selected miRNAs (miR-19b-3p and miR-874) were accordant with microarray data compared between the two activating mutations. (Figure 2). The differential expression of miR-19b-3p detected by qRT-PCR showed statistical significance between two activating mutations patients.

## DISCUSSION

Del19 and L858R are gradually being characterized as two distinct mutations with different clinical outcome. However, up to date, no mechanism underlying the difference has been reported. Our original goal was to detect miRNAs and genes differently altered in the two mutants which could partly answer the question. Of all miRNAs on the chip, we finally identified 79 miRNAs with fold > 5. Three major networks were detected and merged via overlapping genes. Network 1 and 2 included genes involved in organismal injury, abnormalities and cancer. Network 3 mainly connected developmental disorder, hereditary disorder, skeletal and muscular disorders.

There are many reports on how the mutations abnormally activate EGFR and their hypersensitivity with TKI. Direct binding measurements show that gefitinib binds 20-fold more tightly to the L858R mutant than the wild-type enzyme [13]. Besides of the fact that L858R is not constitutively in the active conformation [14], it even functions when coexisting with drug resistant mutation [15]. One more explanation for its abnormally high activity is by promoting EGFR dimerization rather than allowing activation independently [16].

Dominant genes in network 1 are MYC, AGO2, YBX1, CCNE1 and interestingly, tretinoin. MYC plays an extremely important role in cell cycle progression, apoptosis and cellular transformation [17]. In addition, it has a more specific relationship with lung cancer [18]. AGO2 is vital in RNA silencing process as essential catalytic components of the RNA-induced silencing complex that binds miRNA as guide to specific targets through base pairing to initiate mRNA cleavage or translation inhibition [19, 20]. Since it also functions in inhibiting cell and tumor growth [21], it may be worth investigating. YBX1 was basically altered by AGO2 and MYC with variant relationships with cyclin D1, p53 and other tumor-associated factors [22–24]. CCNE1 functions in initiation of cell proliferation, indicating that the process is probably different between the two mutations. Surprisingly, tretinoin directly regulates almost every important gene presented above.

Network 2 has three important factors connecting almost all the other components. Vascular endothelial growth factor (VEGF) was honored to be a landmark in oncology [25]. Only since then did people turn to focus on the simple fact that angiogenesis is essential to tumor development. This factor affects tumor vasculature in different ways, allowing the vessels to establish, to grow and to survive [26]. Since VEGF is continuously and highly expressed across wide range of solid tumors, relative drugs have shown beneficial to patients, including those with lung cancer. However, the benefit is complex and unstable, probably involves multiple mechanisms. Insulin not only connects with VEGF and Akt, but also some of the aberrant miRNAs directly. So could it be hypothesized that the metabolism of carbohydrates and fats is connected with the main topic? Or how will insulin influence the benefit? Akt inhibition decreases release of intact insulin proteins in mouse. Akt also functions in cellular pathways, survival for example. Several differently expressed miRNAs including miR-19b-3p identified in this study modulate directly their target Akt and can be involved in Akt-VEGF signaling pathway. As Akt is not the termination of any functional path, further analysis is needed to explore the downstream effect.

Network 3 mainly covered developmental disorders. Being one of the most earliest found oncogenes, P53 is really powerful responding to diverse cellular stresses to regulate target genes that induce cell cycle arrest, apoptosis, senescence, DNA repair and changes in metabolism. In addition, p53 could induce apoptosis through non-transcriptional cytoplasmic processes. What's more, P53 enhances posttranscriptional maturation of several miRNAs and directly affects EGFR transcription [27, 28]. Since P53 is frequently mutated or inactivated in all types of lung cancer, the mechanism is still too complicated to clarify so far. Follicle-stimulating hormone (FSH), was known basically in reproduction. However, FSH receptor is expressed by the vascular endothelium in a wide range of human tumors as well. FSH receptor was also found in the majority of metastatic tumors. [29, 30] Coincidentally, FSH also has the ability to up-regulate VEGF to produce angiogenic factors upon vessel maturation; whereas, it also possess the potential to promote p53-induced apoptosis indicated by reduction of insulin-like growth factor binding protein (IGFBP3) [31]. Insulin-like growth factor 1 receptor (IGF1R) is an important molecule involving the tumor immunity. A meta-analysis suggested IGF1R positive expression as an unfavorable factor associated with smoking status and tumor size for disease free survival in NSCLC patients [32]. This receptor also connects with hypoxia closely for that hypoxia induces epithelial-mesenchymal transition in NSCLC cells through activation of IGF1R [33]. Furthermore, hypoxia increases the population of lung cancer stem cell resistant to gefitinib in EGFR mutation-positive NSCLC by activating IGF1R [34]. In a phase I/II randomized trial, the addition of dalotuzumab (a selective anti-IGF1R antibody) to erlotinib did not improve efficacy outcome in patients with refractory advanced NSCLC [35]. However, in a later study, IGF1R knockdown not only significantly sensitizes resistant cells to afatinib, but also induces apoptosis in afatinib resistance cells. In addition, combination treatment with afatinib and linsitinib shows more than additive effects on tumor growth [36]. A recent research also showed that activation of IGF1R plays a critical role in radio resistance of lung cancer cell through p53 pathway [37]. Therefore, anti-IGF1R therapy remains controversial. Ephrin type-B receptor 6 (EPHB6) influences cell adhesion and migration, the expression of which is usually down regulated during tumor progression. Data shows EPHB6 is a metastasis inhibitory gene frequently silenced by hypermethylation in NSCLC [38]. However, EPHB6 mutations were also proved to promote metastasis in a subset of patients with non-small cell lung cancer [39]. Calcifediol merges in both network 2 and 3, directly affecting some of the differently expressed miRNAs and was associated with improved survival in NSCLC [40, 41].

In conclusion, the expression of key regulators such as MYC, TP53 and VEGF could be important in distinguishing the relevant mechanisms. Genetic mutation or deletion leads to the inactivation of tumor suppressor gene in the carcinogenesis. Furthermore, miRNAs modify tumor suppressor genes at transcriptional level and suppress their functions in response of TKI [42].

Surely the problem has still too much space to cover. For example, ethnicity, higher prevalence of de novo T790M-resistant mutation in L858R, differential EGF-induced tyrosine phosphorylation patterns in L858R or even L858R-mutated patients respond better on chemotherapy. Therefore, future studies are needed to examine these in detail and will provide insight into their roles in NSCLC even lung cancer treatment.

## MATERIALS AND METHODS

### Sample collection

This study was conducted in line with the Helsinki Declaration and approved by the Institutional Review Board of the First Affiliated Hospital of Xiamen University. Prior to TKI treatment, peripheral blood (8 ml) were collected from 22 NSCLC patients harboring either del19 (11 patients) or L858R (11 patients) from August 2013 to February 2016. All patients were pathologically confirmed lung adenocarcinoma and tested EGFR mutation on tumor specimens. Clinical staging was determined according to the American Joint Committee on Cancer (AJCC) TNM classification.

### RNA extraction

Plasma was separated within half an hour with two centrifuging steps (850 g/10 min and 2400 g/10 min) at 4°C. Total RNA was isolated from 200 μl plasma. Total RNA was isolated using TRIzol (Invitrogen) and purified with RNeasy mini kit (QIAGEN) according to manufacturer's instructions. RNA quality and quantity was measured by using nanodrop spectrophotometer (ND-1000, Nanodrop Technologies) and RNA Integrity was determined by gel electrophoresis.

### MiRNA labeling and array hybridization

RNA labeling and array hybridization was according to Exiqon's manual. After quality control, the miRCURY^™^ Hy3^™^/Hy5^™^ Power labeling kit (Exiqon, Vedbaek, Denmark) was used according to the manufacturer's guideline for miRNA labelling by following steps: 1 μL RNA in 2.0 μL of water was combined with 1.0 μL of CIP buffer and CIP (Exiqon). The mixturewas incubated for 30 min at 37°C. The Reaction was terminated by incubation for 5 min at 95°C. Then 3.0 μL of labeling buffer, 1.5 μL of fluorescent label (Hy3TM), 2.0 μL of DMSO, 2.0 μL of labeling enzyme were added into the mixture. The labelingreaction was incubated for 1 h at 16°C Terminated by incubation for 15 min at 65°C. After stopping the labeling procedure, the Hy3^™^-labeled samples were hybridized on the miRCURYTM LNA Array (v.18.0) (Exiqon) according to array manual. The total 25 μL mixture from Hy3^™^-labeled samples with 25 μL hybridization buffer were first denatured for 2 min at 95°C, incubated on ice for 2 min. Then hybridized to the microarray for 16–20 h at 56°C in a 12-Bay Hybridization Systems (Hybridization System - Nimblegen Systems, Inc., Madison, WI, USA). Following hybridization, the slides were achieved, washed several times using Wash buffer kit (Exiqon). Then the slides were scanned using the Axon GenePix 4000B microarray scanner (Axon Instruments, Foster City, CA).

### Array data analysis

Scanned images were then imported into GenePix Pro 6.0 software for grid alignment and data extraction. Replicated miRNAs were averaged and miRNAs with intensity > = 30 in all samples were chosen for calculating normalization factor. After normalization using the Median normalization factor, significant differentially expressed miRNAs between two groups were identified through Fold change and *P*-value.

### Validation with quantitative real-time PCR

Validation of candidate miRNAs was performed using SYBR green mixtures after reverse-transcription. Cel-mir-39 added in the process of miRNA purification from plasma was used as the internal control. The PCR reaction was evaluated by melting curve analysis and the calculations for determining the relative level of miRNA expression were made using the cycle threshold (Ct) method. The mean Ct values from triplicate measurements were used to calculate relative expression of the target miRNAs with normalization to cel-mir-39 using the 2-ΔCt method.

### Network and gene ontology analyses

Genetic networks and functional classification of differentially expressed miRNAs were investigated with IPA (Ingenuity Systems, Mountain View, CA), a web delivered tool that enables the discovery, visualization, and exploration of molecular interaction networks in gene expression data [43].

### Statistical analysis

The comparison of different miRNA expression in plasma between del19 and L858R mutations was analyzed using the Students *t*-test. The Fisher's test was used to analyze the significance of the genetic networks identified by the IPA tool. A *p* value < 0.05 was considered statistically significant.

## SUPPLEMENTARY MATERIALS





## References

[1] Siegel RL, Miller KD, Jemal A (2015). Cancer statistics, 2015. CA Cancer J Clin.

[2] Breathnach OS, Freidlin B, Conley B, Green MR, Johnson DH, Gandara DR, O'Connell M, Shepherd FA, Johnson BE (2001). Twenty-two years of phase III trials for patients with advanced non-small-cell lung cancer: sobering results. J Clin Oncol.

[3] Shigematsu H, Lin L, Takahashi T, Nomura M, Suzuki M, Wistuba II, Fong KM, Lee H, Toyooka S, Shimizu N, Fujisawa T, Feng Z (2005). Clinical and biological features associated with epidermal growth factor receptor gene mutations in lung cancers. J Natl Cancer Inst.

[4] Tokumo M, Toyooka S, Kiura K, Shigematsu H, Tomii K, Aoe M, Ichimura K, Tsuda T, Yano M, Tsukuda K, Tabata M, Ueoka H, Tanimoto M (2005). The relationship between epidermal growth factor receptor mutations and clinicopathologic features in non-small cell lung cancers. Clin Cancer Res.

[5] Hirsch FR (2006). Molecular predictors of outcome with gefitinib in a phase III placebo-controlled study in advanced non-small-cell lung cancer. J Clin Oncol.

[6] Jackman DM, Yeap BY, Sequist LV, Lindeman N, Holmes AJ, Joshi VA, Bell DW, Huberman MS, Halmos B, Rabin MS, Haber DA, Lynch TJ, Meyerson M (2006). Exon 19 deletion mutations of epidermal growth factor receptor are associated with prolonged survival in non-small cell lung cancer patients treated with gefitinib or erlotinib. Clin Cancer Res.

[7] Riely GJ (2006). Clinical course of patients with non-small cell lung cancer and epidermal growth factor receptor exon 19 and exon 21 mutations treated with gefitinib or erlotinib. Clin Cancer Res.

[8] Maemondo M, Inoue A, Kobayashi K, Sugawara S, Oizumi S, Isobe H, Gemma A, Harada M, Yoshizawa H, Kinoshita I, Fujita Y, Okinaga S, Hirano H (2010). Gefitinib or chemotherapy for non-small-cell lung cancer with mutated EGFR. N Engl J Med.

[9] Mitsudomi T, Morita S, Yatabe Y, Negoro S, Okamoto I, Tsurutani J, Seto T, Satouchi M, Tada H, Hirashima T, Asami K, Katakami N, Takada M (2010). Gefitinib versus cisplatin plus docetaxel in patients with non-small-cell lung cancer harbouring mutations of the epidermal growth factor receptor (WJTOG3405): an open label, randomised phase 3 trial. Lancet Oncol.

[10] Zhou C, Wu Y-L, Chen G, Feng J, Liu X-Q, Wang C, Zhang S, Wang J, Zhou S, Ren S, Lu S, Zhang L, Hu C Erlotinib versus chemotherapy as first-line treatment for patients with advanced EGFR mutation-positive non-small-cell lung cancer (OPTIMAL, CTONG-0802): a multicentre, open-label, randomised, phase 3 study. The Lancet Oncology.

[11] Calin GA, Croce CM (2006). MicroRNA signatures in human cancers. Nat Rev Cancer.

[12] Cummins JM, Velculescu VE (2006). Implications of micro-RNA profiling for cancer diagnosis. Oncogene.

[13] Yun CH, Boggon TJ, Li Y, Woo MS, Greulich H, Meyerson M, Eck MJ (2007). Structures of lung cancer-derived EGFR mutants and inhibitor complexes: mechanism of activation and insights into differential inhibitor sensitivity. Cancer Cell.

[14] Gajiwala KS, Feng J, Ferre R, Ryan K, Brodsky O, Weinrich S, Kath JC, Stewart A (2013). Insights into the aberrant activity of mutant EGFR kinase domain and drug recognition. Structure.

[15] Oikawa T, Ohira T, Otani K, Hagiwara M, Konaka C, Ikeda N (2015). Clinical usefulness of gefitinib for non-small-cell lung cancer with a double epidermal growth factor receptor mutation. Mol Clin Oncol.

[16] Shan Y, Eastwood MP, Zhang X, Kim ET, Arkhipov A, Dror RO, Jumper J, Kuriyan J, Shaw DE (2012). Oncogenic mutations counteract intrinsic disorder in the EGFR kinase and promote receptor dimerization. Cell.

[17] Finver SN, Nishikura K, Finger LR, Haluska FG, Finan J, Nowell PC, Croce CM (1988). Sequence analysis of the MYC oncogene involved in the t(8;14)(q24;q11) chromosome translocation in a human leukemia T-cell line indicates that putative regulatory regions are not altered. Proc Natl Acad Sci USA.

[18] Soucek L, Whitfield J, Martins CP, Finch AJ, Murphy DJ, Sodir NM, Karnezis AN, Swigart LB, Nasi S, Evan GI (2008). Modelling Myc inhibition as a cancer therapy. Nature.

[19] Hannon GJ (2002). RNA interference. Nature.

[20] Meister G, Landthaler M, Patkaniowska A, Dorsett Y, Teng G, Tuschl T Human Argonaute2 Mediates RNA Cleavage Targeted by miRNAs and siRNAs. Molecular Cell.

[21] Zhang X, Graves P, Zeng Y (2013). Overexpression of human Argonaute2 inhibits cell and tumor growth. Biochim Biophys Acta.

[22] Okamoto T, Izumi H, Imamura T, Takano H, Ise T, Uchiumi T, Kuwano M, Kohno K (2000). Direct interaction of p53 with the Y-box binding protein, YB-1: a mechanism for regulation of human gene expression. Oncogene.

[23] Harada M, Kotake Y, Ohhata T, Kitagawa K, Niida H, Matsuura S, Funai K, Sugimura H, Suda T, Kitagawa M (2014). YB-1 promotes transcription of cyclin D1 in human non-small-cell lung cancers. Genes Cells.

[24] Lasham A, Samuel W, Cao H, Patel R, Mehta R, Stern JL, Reid G, Woolley AG, Miller LD, Black MA, Shelling AN, Print CG, Braithwaite AW (2012). YB-1, the E2F pathway, and regulation of tumor cell growth. J Natl Cancer Inst.

[25] Folkman J, Merler E, Abernathy C, Williams G (1971). Isolation of a tumor factor responsible for angiogenesis. J Exp Med.

[26] Folkman J (1995). Angiogenesis in cancer, vascular, rheumatoid and other disease. Nat Med.

[27] Bheda A, Creek KE, Pirisi L (2008). Loss of p53 induces epidermal growth factor receptor promoter activity in normal human keratinocytes. Oncogene.

[28] Suzuki HI, Yamagata K, Sugimoto K, Iwamoto T, Kato S, Miyazono K (2009). Modulation of microRNA processing by p53. Nature.

[29] Radu A, Pichon C, Camparo P, Antoine M, Allory Y, Couvelard A, Fromont G, Hai MT, Ghinea N (2010). Expression of follicle-stimulating hormone receptor in tumor blood vessels. N Engl J Med.

[30] Siraj A, Desestret V, Antoine M, Fromont G, Huerre M, Sanson M, Camparo P, Pichon C, Planeix F, Gonin J, Radu A, Ghinea N (2013). Expression of follicle-stimulating hormone receptor by the vascular endothelium in tumor metastases. BMC Cancer.

[31] Sasson R, Dantes A, Tajima K, Amsterdam A (2003). Novel genes modulated by FSH in normal and immortalized FSH-responsive cells: new insights into the mechanism of FSH action. Faseb j.

[32] Zhao S, Qiu Z, He J, Li L, Li W (2014). Insulin-like growth factor receptor 1 (IGF1R) expression and survival in non-small cell lung cancer patients: a meta-analysis. International Journal of Clinical and Experimental Pathology.

[33] Nurwidya F, Takahashi F, Kobayashi I, Murakami A, Kato M, Minakata K, Nara T, Hashimoto M, Yagishita S, Baskoro H, Hidayat M, Shimada N, Takahashi K (2014). Treatment with insulin-like growth factor 1 receptor inhibitor reverses hypoxia-induced epithelial-mesenchymal transition in non-small cell lung cancer. Biochem Biophys Res Commun.

[34] Murakami A, Takahashi F, Nurwidya F, Kobayashi I, Minakata K, Hashimoto M, Nara T, Kato M, Tajima K, Shimada N, Iwakami S-i, Moriyama M, Moriyama H (2014). Hypoxia Increases Gefitinib-Resistant Lung Cancer Stem Cells through the Activation of Insulin-Like Growth Factor 1 Receptor. PLoS ONE.

[35] Moran T, Felip E, Keedy V, Borghaei H, Shepherd FA, Insa A, Brown H, Fitzgerald T, Sathyanarayanan S, Reilly JF, Mauro D, Hsu K, Yan L (2014). Activity of dalotuzumab, a selective anti-IGF1R antibody, in combination with erlotinib in unselected patients with Non-small-cell lung cancer: a phase I/II randomized trial. Exp Hematol Oncol.

[36] Lee Y, Wang Y, James M, Jeong JH, You M (2016). Inhibition of IGF1R signaling abrogates resistance to afatinib (BIBW2992) in EGFR T790M mutant lung cancer cells. Mol Carcinog.

[37] Zhang H, Zhang C, Wu D (2015). Activation of insulin-like growth factor 1 receptor regulates the radiation-induced lung cancer cell apoptosis. Immunobiology.

[38] Yu J, Bulk E, Ji P, Hascher A, Tang M, Metzger R, Marra A, Serve H, Berdel WE, Wiewroth R, Koschmieder S, Muller-Tidow C (2010). The EPHB6 receptor tyrosine kinase is a metastasis suppressor that is frequently silenced by promoter DNA hypermethylation in non-small cell lung cancer. Clin Cancer Res.

[39] Bulk E, Yu J, Hascher A, Koschmieder S, Wiewrodt R, Krug U, Timmermann B, Marra A, Hillejan L, Wiebe K, Berdel WE, Schwab A, Muller-Tidow C (2012). Mutations of the EPHB6 receptor tyrosine kinase induce a pro-metastatic phenotype in non-small cell lung cancer. PLoS One.

[40] Srinivasan M, Parwani AV, Hershberger PA, Lenzner DE, Weissfeld JL (2011). Nuclear vitamin D receptor expression is associated with improved survival in non-small cell lung cancer. J Steroid Biochem Mol Biol.

[41] Kim SH, Chen G, King AN, Jeon CK, Christensen PJ, Zhao L, Simpson RU, Thomas DG, Giordano TJ, Brenner DE, Hollis B, Beer DG, Ramnath N (2012). Characterization of vitamin D receptor (VDR) in lung adenocarcinoma. Lung Cancer.

[42] Donzelli S, Cioce M, Muti P, Strano S, Yarden Y, Blandino G (2016). MicroRNAs: Non-coding fine tuners of receptor tyrosine kinase signalling in cancer. Semin Cell Dev Biol.

[43] Calvano SE, Xiao W, Richards DR, Felciano RM, Baker HV, Cho RJ, Chen RO, Brownstein BH, Cobb JP, Tschoeke SK, Miller-Graziano C, Moldawer LL, Mindrinos MN (2005). A network-based analysis of systemic inflammation in humans. Nature.

