# Supplementary material for: Plasma MiRNA alterations between NSCLC patients harboring Del19 and L858R EGFR mutations
Source: Oncotarget. 2016 Jul 24;7(34):54965–72. doi: 10.18632/oncotarget.10829 (PMC5342394; doi:10.18632/oncotarget.10829)
Supplement: Supplementary file 1 [file oncotarget-07-54965-s001.pdf]

# Plasma MiRNA alterations between NSCLC patients harboring Del19 and L858R EGFR mutations

**Supplementary Materials**

**Supplementary Table S1: Differentially expressed miRNAs of microarray analysis.**
See Supplementary_Table_S1.
