# Supplementary material for: Plasma MiRNA alterations between NSCLC patients harboring Del19 and L858R EGFR mutations
Source: Oncotarget. 2016 Jul 24;7(34):54965–72. doi: 10.18632/oncotarget.10829 (PMC5342394; doi:10.18632/oncotarget.10829)
Supplement: Supplementary file 2 [file oncotarget-07-54965-s002.doc]

**Supplement 1**

**Differentially expressed miRNAs of microarray analysis**

| **Name** | **Fold** | **M19** | **M21** |
| --- | --- | --- | --- |
| hsa-miR-4326 | 27.09236 | 0.17942 | 0.006623 |
| hsa-miR-1208 | 25.88655 | 0.162809 | 0.006289 |
| hsa-let-7c-3p | 25.51547 | 0.168977 | 0.006623 |
| hsa-miR-4774-3p | 25.14144 | 0.158122 | 0.006289 |
| hsa-miR-4681 | 24.26706 | 0.160709 | 0.006623 |
| hsa-miR-4458 | 22.85926 | 0.151386 | 0.006623 |
| hsa-miR-328-3p | 18.74302 | 0.117881 | 0.006289 |
| hsa-miR-5706 | 17.71006 | 0.117285 | 0.006623 |
| hsa-miR-4529-3p | 17.38835 | 0.230309 | 0.013245 |
| hsa-miR-2682-5p | 16.54734 | 0.21917 | 0.013245 |
| hsv2-miR-H4-5p | 15.5784 | 0.206336 | 0.013245 |
| hsa-miR-628-5p | 14.93851 | 0.098931 | 0.006623 |
| hsa-miR-874 | 14.62109 | 0.453488 | 0.031016 |
| hsa-miR-21-3p | 13.20833 | 0.174945 | 0.013245 |
| hsa-miR-1262 | 13.06971 | 0.168754 | 0.012912 |
| hsa-miR-5701 | 12.84761 | 0.161605 | 0.012579 |
| hsa-miR-381-5p | 12.79617 | 0.160958 | 0.012579 |
| hsv1-miR-H14-5p | 12.42426 | 0.31068 | 0.025006 |
| hsa-miR-455-5p | 10.46965 | 0.265132 | 0.025324 |
| hsa-miR-372-5p | 10.19832 | 0.320702 | 0.031447 |
| hsa-miR-3654 | 10.13935 | 0.201444 | 0.019868 |
| hsa-miR-501-3p | 9.784884 | 0.052326 | 0.005348 |
| hsa-miR-4295 | 9.682692 | 0.182692 | 0.018868 |
| hsa-miR-4417 | 9.606083 | 0.127233 | 0.013245 |
| hsa-miR-152-5p | 9.438677 | 0.100948 | 0.010695 |
| hsa-miR-9-3p | 9.398576 | 0.118221 | 0.012579 |
| hsa-miR-4482-3p | 9.378482 | 0.176952 | 0.018868 |
| hsa-miR-196a-3p | 8.626224 | 0.271265 | 0.031447 |
| hsa-miR-506-5p | 8.381835 | 0.166527 | 0.019868 |
| hsa-miRPlus-C1110 | 8.124553 | 0.10761 | 0.013245 |
| hsa-miR-4524a-3p | 7.93915 | 0.199727 | 0.025157 |
| hsa-miR-4512 | 7.899502 | 0.149047 | 0.018868 |
| ebv-miR-BART11-5p | 7.780734 | 0.432803 | 0.055625 |
| hsv1-miR-H4-5p | 7.73713 | 0.124125 | 0.016043 |
| hsa-miR-323b-3p | 7.722674 | 0.082595 | 0.010695 |
| hsa-miR-4423-5p | 7.515227 | 0.094531 | 0.012579 |
| hsa-miR-549a | 7.346619 | 0.078573 | 0.010695 |
| hsv2-miR-H2 | 7.3046 | 0.078124 | 0.010695 |
| hsa-miR-376a-5p | 7.147945 | 0.269734 | 0.037736 |
| hsa-miR-645 | 7.119394 | 0.208609 | 0.029302 |
| hsa-miR-5047 | 7.118182 | 0.188561 | 0.02649 |
| hsa-miR-216a-5p | 7.038189 | 0.111836 | 0.01589 |
| hsa-miR-4797-3p | 7 | 0.037433 | 0.005348 |
| hsa-miR-370-5p | 6.909532 | 0.183034 | 0.02649 |
| hsa-miR-526b-3p | 6.595398 | 0.128837 | 0.019534 |
| ebv-miR-BART13-5p | 6.525028 | 0.10468 | 0.016043 |
| hsa-miR-4680-3p | 6.467121 | 0.203369 | 0.031447 |
| hsa-miR-34b-3p | 6.255944 | 0.236073 | 0.037736 |
| hsa-miR-1273c | 6 | 0.096257 | 0.016043 |
| hsa-miR-202-3p | 5.997917 | 0.135027 | 0.022512 |
| hsa-miR-379-5p | 5.996376 | 0.231273 | 0.038569 |
| hsa-miR-3200-3p | 5.985451 | 0.112933 | 0.018868 |
| hsa-miR-4677-5p | 5.87064 | 0.062788 | 0.010695 |
| hsa-miR-2110 | 5.815034 | 0.192551 | 0.033113 |
| hsa-miR-23b-5p | 5.757022 | 0.266882 | 0.046358 |
| hsa-miR-27b-5p | 5.699291 | 0.264206 | 0.046358 |
| hsa-miR-1260a | 5.694918 | 0.107451 | 0.018868 |
| hsa-miR-4433a-5p | 5.653289 | 0.248887 | 0.044025 |
| hsa-miR-3660 | 5.623102 | 0.106096 | 0.018868 |
| hsv1-miR-H16 | 5.486694 | 0.088022 | 0.016043 |
| hsa-miR-3121-5p | 5.468566 | 0.198275 | 0.036257 |
| hsa-miR-210-3p | 5.435973 | 0.211471 | 0.038902 |
| hsa-miR-5190 | 5.409942 | 0.190742 | 0.035258 |
| hsa-miR-28-5p | 5.3645 | 0.086061 | 0.016043 |
| hsa-miR-4682 | 5.346967 | 0.30444 | 0.056937 |
| hsa-miR-5703 | 5.292377 | 0.084904 | 0.016043 |
| hsa-miR-766-3p | 5.268063 | 0.099397 | 0.018868 |
| hsa-miR-4492 | 5.203488 | 0.083478 | 0.016043 |
| hsa-miR-552-3p | 5.168381 | 0.747942 | 0.144715 |
| hsa-miR-3130-3p | 5.16136 | 0.055202 | 0.010695 |
| hsa-miR-429 | 5.158342 | 0.309922 | 0.060082 |
| hsa-miRPlus-G1065-5p | 5.155864 | 0.06829 | 0.013245 |
| hsa-miR-4419a | 5.127045 | 0.16977 | 0.033113 |
| hsa-miR-502-3p | 5.112308 | 0.101569 | 0.019868 |
| hsa-miR-519a-3p | 5.064773 | 0.15927 | 0.031447 |
| hsa-miR-18a-5p | 5.010526 | 0.126051 | 0.025157 |
| hsa-miR-19b-3p | 0.16071 | 0.187719 | 1.16806 |
| hsa-miR-761 | 0.072809 | 0.027933 | 0.383648 |
| hsa-miR-5095 | 0.062123 | 0.053527 | 0.861635 |
